# Formation and Antibacterial Mechanisms of Micro-Arc Oxidation Coatings on Ta2 Pure Titanium by Doping with Cu_2_O Nanoparticles

**DOI:** 10.3390/ma19204273

**Published:** 2026-10-09

**Authors:** Xumin Zhang, Huie Hu, Ansheng Cheng, Hao Wang

**Affiliations:** Naval University of Engineering, Wuhan 430033, China

**Keywords:** pure titanium, micro-arc oxidation, Cu_2_O nanoparticles, formation mechanism, antibacterial mechanism

## Abstract

Cu_2_O nanoparticles (0–4 g/L) were incorporated into the electrolyte to fabricate coatings on TA2 pure titanium via micro-arc oxidation. The effects of different Cu_2_O nanoparticle concentrations on the microstructure, antibacterial performance, formation, and antibacterial mechanisms of the coatings were investigated. Microstructural characterization indicated that Cu_2_O nanoparticles were incorporated into the coating through the combined action of electrophoretic forces and micro-discharges. At 4 g/L Cu_2_O, the incorporation of Cu_2_O nanoparticles shifted from uniform incorporation to particle aggregation. Compared with coatings fabricated using 1 and 2 g/L Cu_2_O nanoparticles, those fabricated using 3 and 4 g/L exhibited superior antibacterial efficacy. Copper ion release was the primary antibacterial mechanism. Micro-arc oxidation coatings modified with 3 g/L Cu_2_O nanoparticles achieved the best compromise between microstructural uniformity and antibacterial performance among the tested concentrations.

## 1. Introduction

Titanium and titanium alloys are renowned as marine metals and are widely used in marine engineering equipment, such as pumps, valves, and piping systems on ships, as well as the hulls of deep-sea submersibles [1,2,3]. This widespread use is attributed to their excellent mechanical properties and corrosion resistance. However, titanium and its alloys also exhibit good biocompatibility. When exposed to the marine environment over extended periods, microorganisms and other organisms attach to the surfaces of marine engineering equipment. These organisms form biofilms, which provide favorable colonization conditions for subsequent fouling organisms, thereby leading to severe biofouling issues. Biofouling significantly affects the overall performance, service life, and maintenance costs of the equipment [4,5,6]. Therefore, it is particularly important to impart antibacterial properties to titanium through surface treatment technologies. Currently, organic coatings and superhydrophobic coatings are common antibacterial surface treatment solutions. Both types of coatings possess good antibacterial performance; however, they are prone to peeling [7]. Coatings produced by micro-arc oxidation (MAO) technology have attracted widespread attention owing to their high adhesive strength and ease of operation [8,9]. However, traditional MAO coatings lack sufficient antibacterial capability. A common strategy is to add antibacterial agents to the electrolyte for incorporation during the MAO process, thereby achieving an antibacterial effect [10,11].

Nanoparticles possess a high surface area to volume ratio. This property offers an efficient means for antibacterial activity. Researchers have achieved antibacterial effects in coatings by incorporating nanoparticles into the electrolyte [12,13]. Chen et al. [14] employed MAO to incorporate TiO_2_ nanoparticles into the coating. Upon photocatalysis, these TiO_2_ nanoparticles generate reactive oxygen species (ROS). The ROS promote the degradation of bacterial membranes. Their photocatalytic activity has been demonstrated to confer excellent antibacterial performance. Elham Nikoomanzari et al. [15] modified the surface of Ti6Al4V via MAO and investigated the effect of ZrO_2_ nanoparticle concentration on the antibacterial performance of the coating. The study found that the antibacterial activity of the coating decreased after the addition of ZrO_2_ nanoparticles. This decrease was due to the formation of the ZrTiO_4_ phase, which has lower photocatalytic activity. In contrast, coatings without ZrO_2_ nanoparticles exhibited higher photocatalytic activity and stronger antibacterial performance, because of the presence of TiO_2_ [16]. However, most marine engineering equipment is frequently exposed to dark environments. Under such conditions, traditional photocatalytic antibacterial strategies become ineffective.

Consequently, antibacterial agents that function via ion release mechanisms are often incorporated into coatings. These agents do not require light exposure and achieve antibacterial efficacy through the release of ions [7,17]. Gong et al. [18] incorporated ZnO nanoparticles into a coating. The coating achieved an antibacterial effect against *Staphylococcus aureus* through the release of Zn^2+^. Husak et al. [19] added CuO particles to the electrolyte. Their antibacterial evaluations indicated that CuO influenced microbial adhesion, resulting in a significant reduction in bacterial colonies. Cerchier et al. [20] prepared MAO coatings by adding Cu particles to the electrolyte. When the coating was exposed to the natural environment, it exhibited excellent antibacterial performance through the release of Cu ions.

Cu_2_O is a primary antifouling agent. It is widely used in the marine coatings industry [21,22]. Some researchers have also compared Cu_2_O with other antibacterial agents. They found its antibacterial performance to be superior to that of other nanoparticles. Zhao et al. [23] added Cu_2_O and ZnO nanoparticles to the electrolyte separately. They evaluated the antibacterial performance of the coatings using Escherichia coli. The results indicated that Cu_2_O nanoparticles exhibited better antibacterial efficacy than ZnO nanoparticles. Tang et al. [24] incorporated Cu_2_O nanoparticles into coatings. Their antibacterial testing revealed that TiO_2_/Cu_2_O coatings exhibited superior antibacterial efficacy against *Pseudomonas aeruginosa* compared to TiO_2_ coatings. However, most researchers have focused only on the antibacterial performance of Cu_2_O nanoparticles. The tendency of nanoparticles to agglomerate in the electrolyte, which leads to an uneven coating appearance, and the underlying reasons for this phenomenon have received little attention. Moreover, studies on the formation mechanism of Cu_2_O nanoparticles incorporated into the coating and the corresponding antibacterial mechanism remain lacking.

In this study, Cu_2_O nanoparticles were added to the electrolyte to fabricate coatings on pure titanium surfaces via MAO. The effects of Cu_2_O nanoparticle concentration on the surface morphology, cross-sectional morphology, phase composition, and antibacterial performance of the coatings were investigated. Furthermore, the formation mechanism of the Cu_2_O nanoparticle-doped MAO coatings and the corresponding antibacterial mechanism were explored.

## 2. Experimental

### 2.1. Materials and Mao Coatings Preparation

In this study, TA2 pure titanium with dimensions of 20 mm × 20 mm × 1 mm was selected as the substrate for MAO (KR-WY-F01-15A, Shenzhen Kairui Energy Saving Technology Co., Ltd., Shenzhen, China). The surface of the pure titanium was sequentially sanded with 240, 400, 600, 800, 1000, and 1500 grit sandpaper, then cleaned in an ultrasonic cleaner (SN-QX-32D, Shanghai Pu Instrument Equipment Co., Ltd., Shanghai, China) with ethanol (Sinopharm Chemical Reagent Co., Ltd., Shanghai, China) for 15 min and dried immediately afterward.

**Figure 1:**
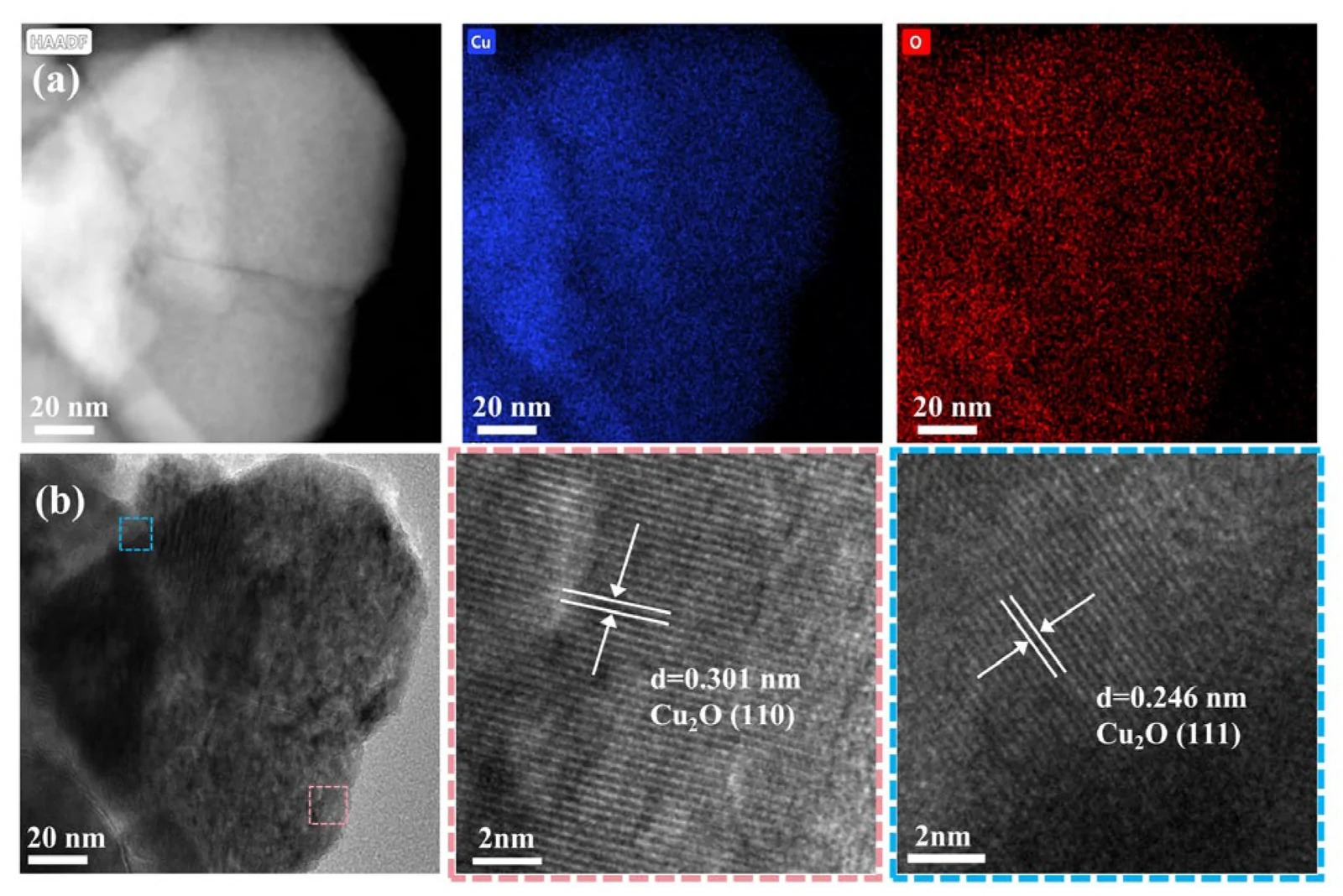
Crystal structure of Cu_2_O nanoparticles: (**a**) EDS elemental maps; (**b**) HR-TEM image.

The MAO process was carried out using a 12 kW AC power supply. The MAO process was performed in constant current mode with a processing time of 25 min, a current density of 5 A/dm^2^, a frequency of 500 Hz, and a duty cycle of 30%. The sample served as the anode, while a double-layer stainless steel electrolytic cell served as the cathode. During the MAO process, a magnetic stirrer (AL-500, Shenzhen Aoling Hengye Technology Co., Ltd., Shenzhen, China) and a cooling system were used to keep the electrolyte temperature below 28 °C. The electrolyte composition consisted of 10 g/L Na_2_SiO_3_, 8 g/L Na_3_PO_4_, 1 g/L NaOH, 1 g/L EDTA-2Na, and 15 g/L sodium citrate. Cu_2_O nanoparticles were subsequently added to this electrolyte. Figure 1 shows the crystal structure of the Cu_2_O nanoparticles. Figure 1a shows the EDS elemental maps, indicating that the nanoparticles primarily contain Cu and O. Figure 1b shows a high-resolution transmission electron microscopy (HR-TEM) image of the Cu_2_O nanoparticles. In this image, lattice fringes with spacings of 0.301 nm and 0.246 nm were clearly visible. These spacings corresponded to the (110) and (111) crystal plane spacings of Cu_2_O, respectively. This observation indicated that the incorporated Cu_2_O nanoparticles were predominantly exposed via the (110) and (111) crystal planes. Cu_2_O nanoparticles were added to the electrolyte at concentrations of 0, 1, 2, 3, and 4 g/L, and the resulting samples were labeled as 0-Cu, 1-Cu, 2-Cu, 3-Cu and 4-Cu.

### 2.2. Characterization

A scanning electron microscope (SEM; Gemini 300, Carl Zeiss, Oberkochen, Germany) equipped with an energy dispersive spectrometer (EDS; Oxford Instruments, High Wycombe, UK) was used to characterize the surface and cross-sectional morphology of the coatings and to perform elemental analysis. The coating thickness was determined via cross-sectional SEM analysis. Cross-sectional transmission electron microscopy (TEM) samples were prepared using a focused ion beam system (FIB; Helios G4 UX, Thermo Fisher Scientific, Hillsboro, OR, USA). High-resolution transmission electron micrographs (HR-TEM) were acquired using a high-resolution transmission electron microscope (TEM; Talos F200X, Thermo Fisher Scientific, Brno, Czech Republic). Phase composition and crystal structure were determined using Fast Fourier Transform (FFT), while elemental distribution was measured using energy-dispersive X-ray spectroscopy surface maps (EDS; Oxford Instruments, High Wycombe, UK). ImageJ software (version 1.54p; National Institutes of Health, Bethesda, MD, USA) was used to perform quantitative analysis of the porosity and pore size of the surface and cross-sections. Phase analysis was performed using an X-ray diffractometer (XRD; DX2100BH, Dandong Haoyuan Instrument Co., Ltd., Dandong, Liaoning, China) with Cu Kα radiation (40 kV, 40 mA), a scan range of 10–80°, a measurement time of 0.24 s, and a step size of 0.02°. An X-ray photoelectron spectrometer (XPS; Thermo Scientific ESCALAB Xi+, Thermo Fisher Scientific, East Grinstead, West Sussex, UK) was used to investigate the chemical states of the elements, with calibration performed using the C 1s peak at a binding energy of 284.8 eV.

### 2.3. Antibacterial Tests

*Staphylococcus aureus* (*S. aureus*) and *Pseudomonas aeruginosa* (*P. aeruginosa*) were selected as test bacteria, representing Gram-positive and Gram-negative bacteria, respectively, to evaluate the antimicrobial performance of the coatings. Bacterial suspensions with concentrations of (1–10) × 10^5^ CFU/mL were prepared using Luria–Bertani (LB) medium. After sterilization, the coated samples were incubated with the bacterial suspensions in a constant-temperature incubator at 37 °C in the dark for 24 h. Subsequently, the bacterial suspension on the coating surface was collected, serially diluted, and 100 μL of the diluted solution was spread onto LB agar plates. Following incubation at 37 °C for 24 h, the resulting bacterial colonies were counted. The antibacterial rate (*R*) was calculated using the following Equation (1) [25]:

(1) $$R=\frac{N_{\mathrm{control}}-N_{\mathrm{sample}}}{N_{\mathrm{control}}}\times 100\%$$

where *N*_control_ and *N*_sample_ represent the number of colonies in the blank control group (Ti) and the coated sample, respectively. For bacterial staining, a 100 μL suspension of *P. aeruginosa* was evenly spread over the sample surface. The samples were then incubated at 37 °C for 24 h. After incubation, the samples were washed twice with sterile PBS. Subsequently, the samples were stained for 15 min using the Live/Dead BacLight Kit (BTN11-220950, Beijing BioLab Technology, Beijing, China). Finally, the stained bacteria were observed under a laser confocal microscope (LSM 800, ZEISS, Oberkochen, Germany). The copper (Cu) ion release concentration from the coating was quantified using an inductively coupled plasma mass spectrometer (ICP-MS; Thermo Scientific, Bremen, Germany). Each sample was statically immersed in 5 mL of PBS solution at 25 ± 2 °C, with an exposed coating area of 4 cm^2^. At predetermined time points, 1 mL of the immersion solution was withdrawn for ICP-MS determination of Cu concentration, and an equal volume of fresh release medium was replenished. The cumulative released concentration of Cu ions was calculated based on the Cu concentrations measured at each sampling time point. All measurements were performed in triplicate.

### 2.4. Statistical Analysis and Reproducibility

At least three independently prepared parallel samples were used for each coating group to ensure experimental reproducibility. For surface porosity and coating thickness, multiple random regions were selected from each sample for analysis. For porosity, at least three SEM images were selected per group, and coating thickness was measured at multiple different positions in cross-sectional SEM images. All quantitative results are expressed as mean ± standard deviation. For the antibacterial tests, three repeated experiments were performed for each group, and the antibacterial rate was calculated based on the number of colony-forming units. For the 4-Cu samples, the antibacterial test was conducted as an overall evaluation without distinguishing between the bright side and the dark side.

## 3. Results and Discussion

### 3.1. Voltage–Time Curves

**Figure 2:**
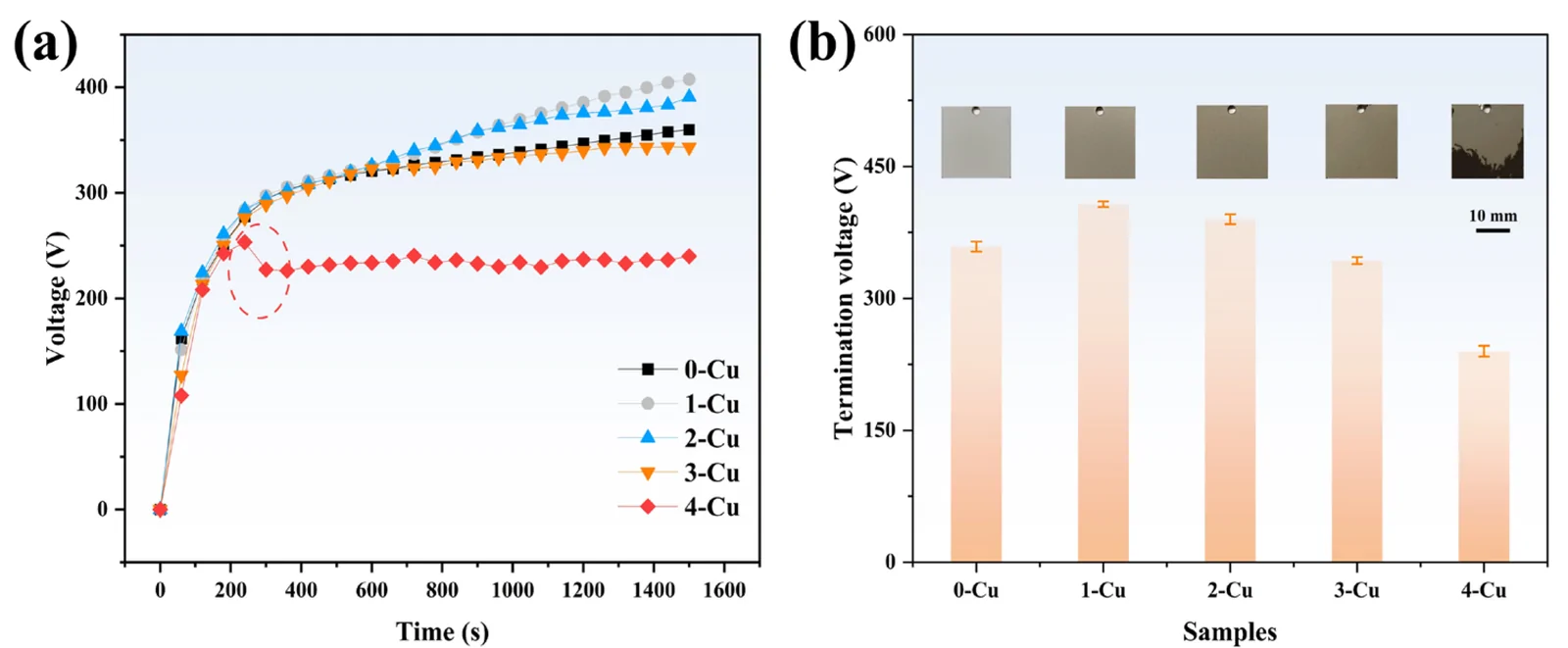
Voltage variation during the MAO process as a function of Cu_2_O nanoparticle concentration: (**a**) voltage–time curves; (**b**) termination voltage and macroscopic photographs of the MAO coatings.

Figure 2a shows the voltage–time curves of Ti during the MAO process in electrolytes with different concentrations of Cu_2_O nanoparticles. The results indicate that, except for the 4-Cu coating, the other samples exhibit similar trends. The applied voltage rises rapidly in the early stages of the MAO process and then increases at a slower rate. The termination voltages of 1-Cu (407 V) and 2-Cu (390 V) are consistently higher than that of the 0-Cu coating (359 V). The termination voltage depends on the concentration of Cu_2_O nanoparticles in the electrolyte. An increase from 1 g/L to 2 g/L leads to a higher termination voltage. For 3 g/L of Cu_2_O nanoparticles, the termination voltage decreased to 343 V. For 4 g/L of Cu_2_O nanoparticles, the trend of the 4-Cu coating curve is different from the trends described above. The 4-Cu coating showed a significant voltage drop at 240 s, with a termination voltage of 240 V, as shown in Figure 2b. This indicated that the addition of an excess of Cu_2_O nanoparticles caused arc instability, thereby disrupting the coating growth process.

The macroscopic photographs in Figure 2b show the MAO coatings formed in electrolytes with different concentrations of Cu_2_O nanoparticles. The 0-Cu coating appears gray, while the coating turns brown upon the addition of Cu_2_O nanoparticles. A higher concentration of Cu_2_O nanoparticles made the brown color deeper. However, the 4-Cu coating prepared with 4 g/L of Cu_2_O nanoparticles has two distinct parts: a brown central area and black edges. This phenomenon is attributed to the concentration of Cu_2_O nanoparticles. The 4-Cu samples from different repeated experiments are shown in Figure S1.

### 3.2. Microstructural and Cross-Section Characterization of Coating Surfaces

**Figure 3:**
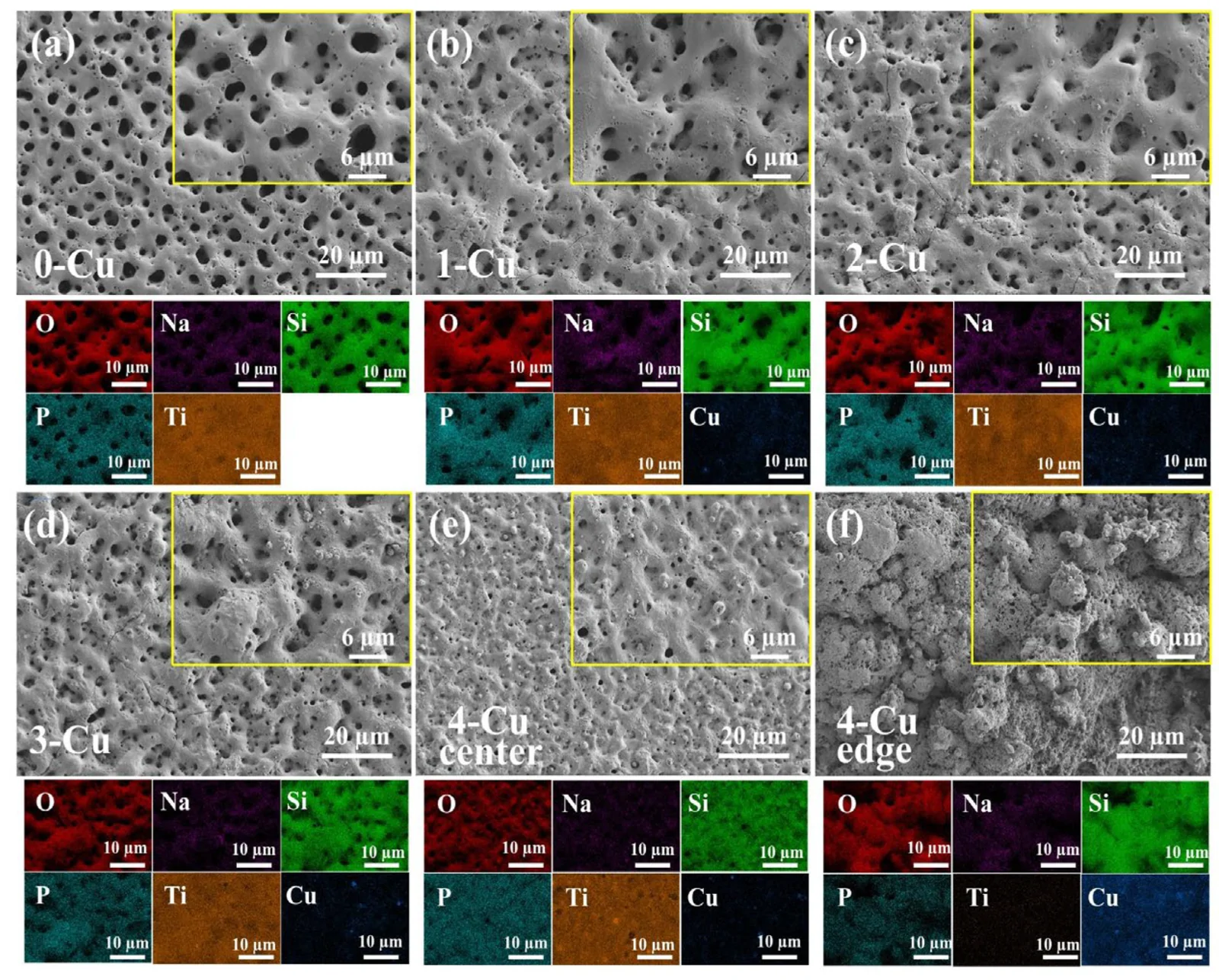
SEM images and EDS surface element distribution of MAO coatings with different concentrations of Cu_2_O nanoparticles: (**a**) 0-Cu, (**b**) 1-Cu, (**c)** 2-Cu, (**d**) 3-Cu, (**e**) 4-Cu center, (**f**) 4-Cu edge.

Figure 3 shows SEM images and EDS surface element distributions of MAO coatings prepared with different concentrations of Cu_2_O nanoparticles. The surface morphology of the 0-Cu coating is shown in Figure 3a, exhibiting a typical MAO coating structure [26]. The coating surface displayed volcanic crater-shaped pores of varying sizes, which served as discharge channels. Figure 3b–f show the surface morphologies of the coatings after the addition of Cu_2_O nanoparticles. During the MAO process, Cu_2_O nanoparticles in the electrolyte migrated toward the anode due to the combined effects of the electric field force and mechanical stirring. Some of these nanoparticles were drawn into the discharge channels, where they melted together with the substrate and oxide film under transient high-temperature and high-pressure conditions, thereby blocking the discharge channels and reducing porosity. When the Cu_2_O nanoparticle concentration was increased to 4 g/L, the central brown region of the 4-Cu coating still exhibited micron-sized pores (Figure 3e). The black marginal region of the 4-Cu coating showed numerous deposits on the surface (Figure 3f).

The elements in the MAO coating were analyzed using EDS (Table 1). The electrolyte contains Cu, Si, P, and Na. During the oxidation process, these elements were incorporated into the TiO_2_ coating, resulting in a coating composition consisting of Cu, Si, P, Na, Ti, and O. Table 1 shows the Cu content in the different coatings. For 0-Cu, 1-Cu, 2-Cu, 3-Cu, 4-Cu (central), and 4-Cu (edge), the Cu contents were 0, 1.45 wt.%, 1.66 wt.%, 2.27 wt.%, 1.30 wt.%, and 27.48 wt.%, respectively. As the electrolyte concentration increased (0–3 g/L), the Cu content in the MAO coating increased, indicating that Cu_2_O participated in the MAO reaction and entered the interior of the coating. The non-uniform coating was prepared by adding 4 g/L Cu_2_O nanoparticles. In this coating, the Cu content in the central region (1.30 wt.%) was lower than that of the 3-Cu coating (2.27 wt.%). The edge region contained deposits. The Cu content in the edge region reached as high as 27.48 wt.%. This indicated that Cu_2_O nanoparticles adhered and aggregated extensively at the edges of the sample surface. When the concentration of Cu_2_O nanoparticles is excessively high, the energy generated by spark discharge is insufficient to melt the Cu_2_O nanoparticles that have migrated to the anode. These nanoparticles then attach to the coating surface through adhesion [27]. The results indicated that adding an appropriate concentration of Cu_2_O nanoparticles produced a uniform MAO coating with a high Cu content.

**Figure 4:**
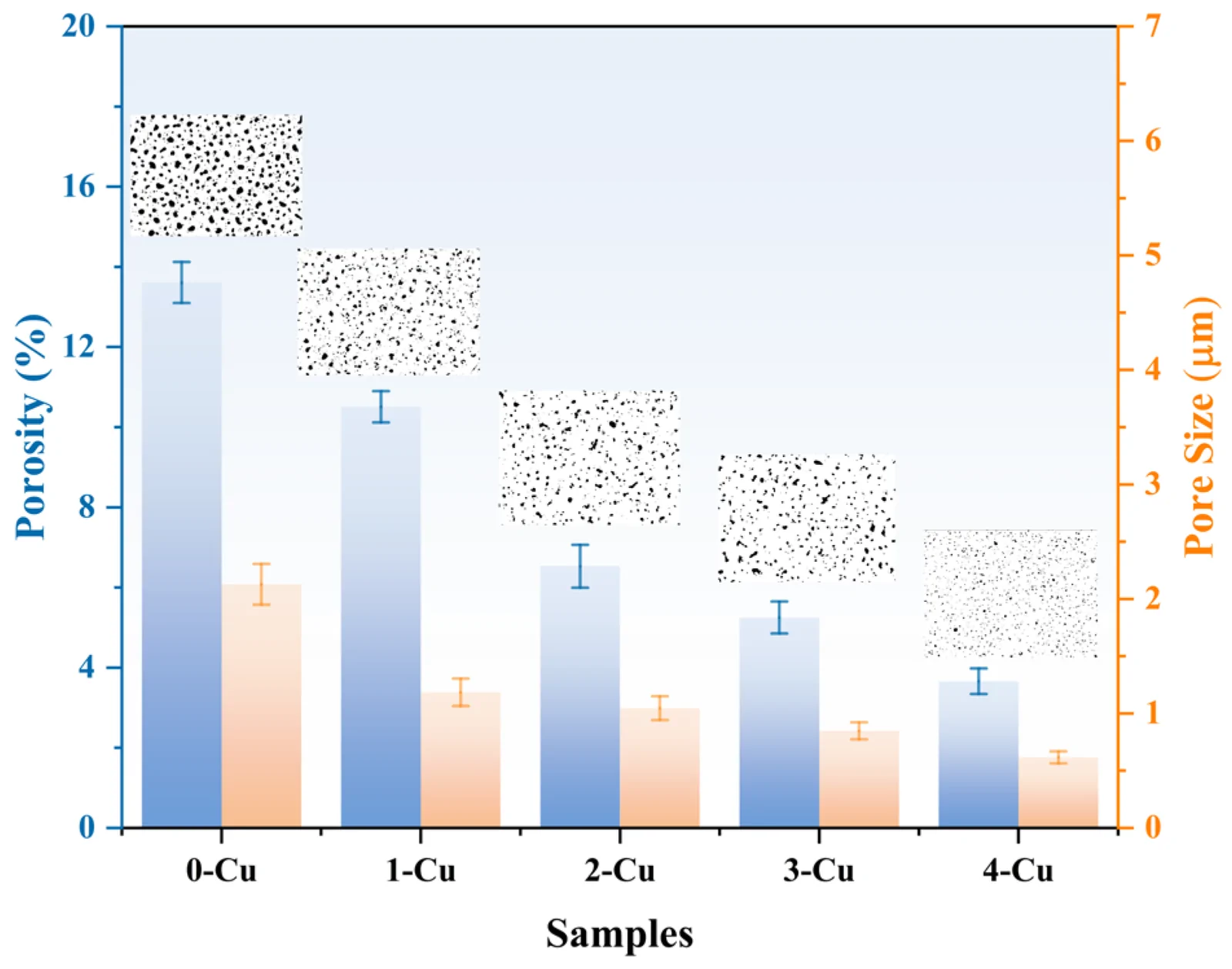
Porosity and pore size analysis of MAO coatings with different concentrations of Cu_2_O nanoparticles.

The porosity and average pore diameter of the coatings were analyzed using Image J software (Figure 4). The 0-Cu coating had an average pore diameter of 2.13 µm and a porosity of 13.61%. After the addition of Cu_2_O nanoparticles, both the porosity and average pore diameter decreased. The 3-Cu coating had an average pore diameter of 0.85 µm and a porosity of 5.25%. The reason is that the high-temperature and high-pressure environment during the MAO process promoted the melting and solidification of the nanoparticles together with the substrate, further increasing the compactness of the coating. When the nanoparticle concentration was further increased to 4 g/L, the average pore diameter was 0.05 µm, and the porosity was 3.66%. The average pore diameter and porosity of the 4-Cu coating decreased further. This was because after deposits adhered to the edges, the discharge became concentrated at the edges. The arc intensity in the central region was significantly weakened. Therefore, the discharge energy decreased and the micro-plasma discharge became milder. As the intensity of individual discharge pulses decreased, less molten material was ejected from the discharge channel. As a result, smaller micropores formed on the surface after solidification.

**Figure 5:**
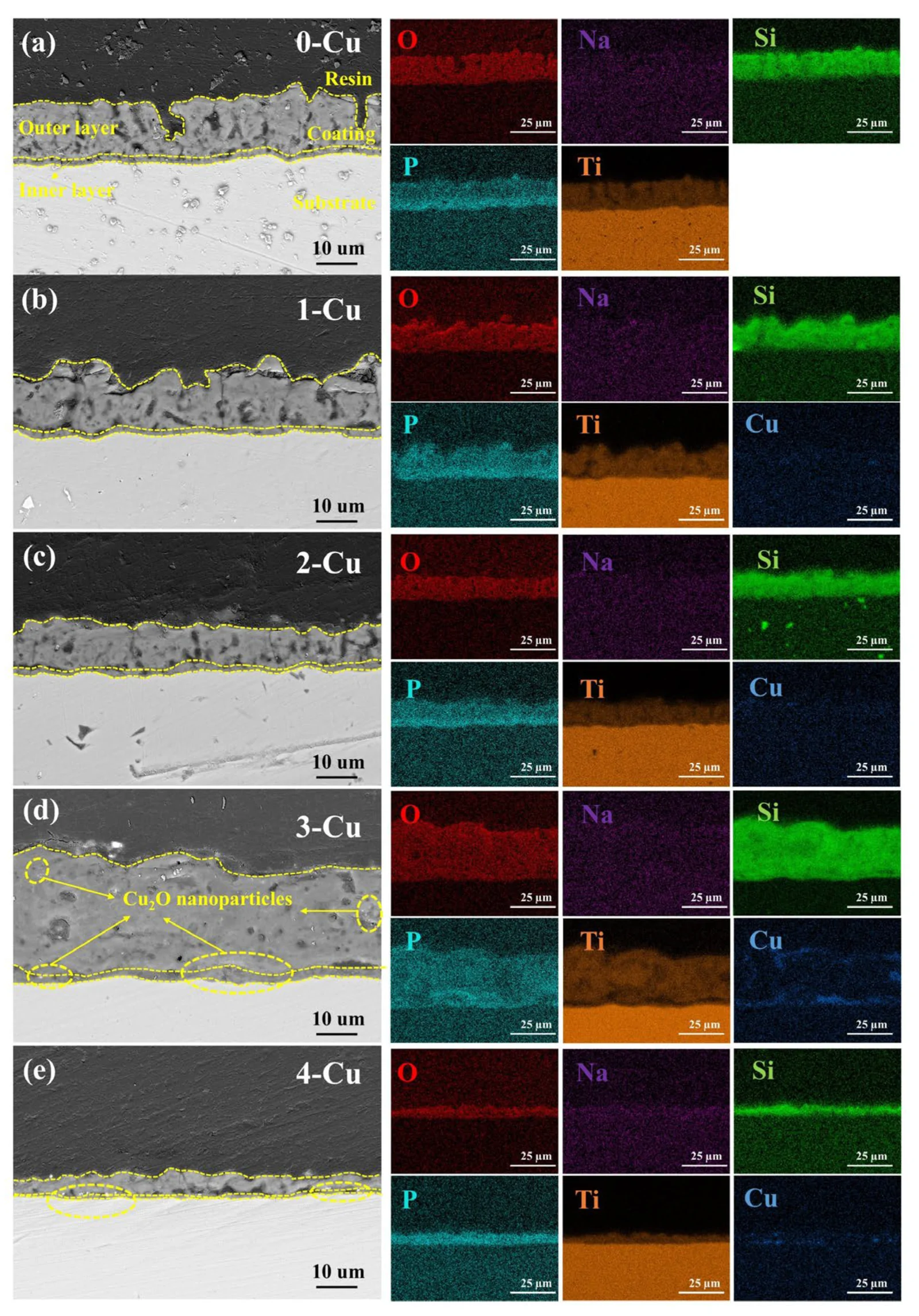
Cross-sectional SEM images and EDS elemental distribution of the MAO coatings: (**a**) 0-Cu; (**b**) 1-Cu; (**c**) 2-Cu; (**d**) 3-Cu; (**e**) 4-Cu.

**Figure 6:**
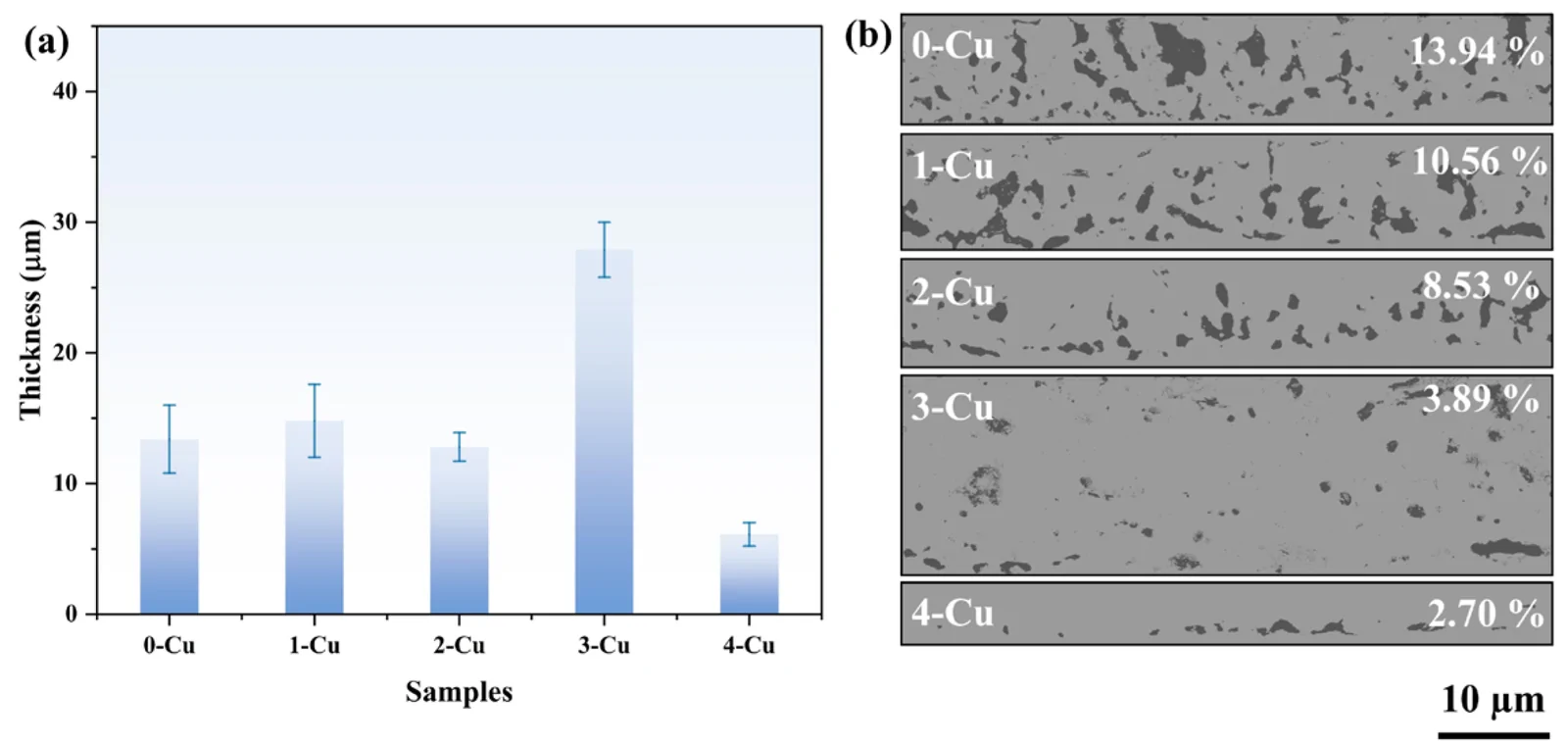
MAO coatings with different concentrations of Cu_2_O nanoparticles: (**a**) coating thickness; (**b**) cross-sectional porosity of the coating.

Figure 5 shows the cross-sectional morphology and elemental distribution of MAO coatings. All coatings exhibited the typical bilayer structure of MAO coatings. This structure consisted of an outer layer and an inner layer. The outer layer was thicker and contained numerous pores. The inner layer was thinner but relatively dense. Figure 6a shows the thickness of MAO coatings prepared with different concentrations of Cu_2_O nanoparticles. The thicknesses of the 0-Cu, 1-Cu, and 2-Cu coatings did not change significantly. The thickness of the 3-Cu coating increased, while that of the 4-Cu coating decreased. This phenomenon can be explained as follows. When an appropriate concentration of Cu_2_O was added, some particles did not melt completely and were deposited directly into the coating. This deposition resulted in a significant increase in thickness. However, a large amount of Cu_2_O nanoparticles was added. Then, particle agglomeration occurred at the edges of the titanium substrate surface (Figure 2b). Sparks concentrated at the edges of the titanium substrate surface. Meanwhile, the spark energy level in the central region of the substrate was low. This low energy led to a reduction in coating thickness. Cu_2_O nanoparticles were observed in the cross-sectional morphologies of 3-Cu and 4-Cu (as highlighted by the yellow circles in Figure 5d,e), but not in those of 1-Cu and 2-Cu. This was due to the low concentration of added Cu_2_O nanoparticles. During the MAO process, the applied voltage was higher (Figure 2a), and the discharge was more intense [28]. The intense discharge caused the nanoparticles to fuse readily with the substrate. Therefore, Cu_2_O nanoparticles were not visible in the cross-sectional morphologies of 1-Cu and 2-Cu. EDS analysis also indicated that Cu was distributed relatively uniformly in 1-Cu and 2-Cu. In contrast, in 3-Cu and 4-Cu, Cu appeared as bright regions primarily at the coating surface and at the metal/oxide interfaces. ImageJ software was used to analyze the cross-sectional morphology and porosity of the coatings. Figure 6b shows the porosity analysis of MAO coatings with different concentrations of Cu_2_O nanoparticles. The porosity of 0-Cu was 13.94%. After the addition of Cu_2_O nanoparticles, the porosity changed significantly. The porosities of 3-Cu and 4-Cu decreased to 3.89% and 2.70%, respectively. A higher concentration of Cu_2_O nanoparticles led to a lower porosity of the coating.

### 3.3. Phase Analysis of the Coatings

**Figure 7:**
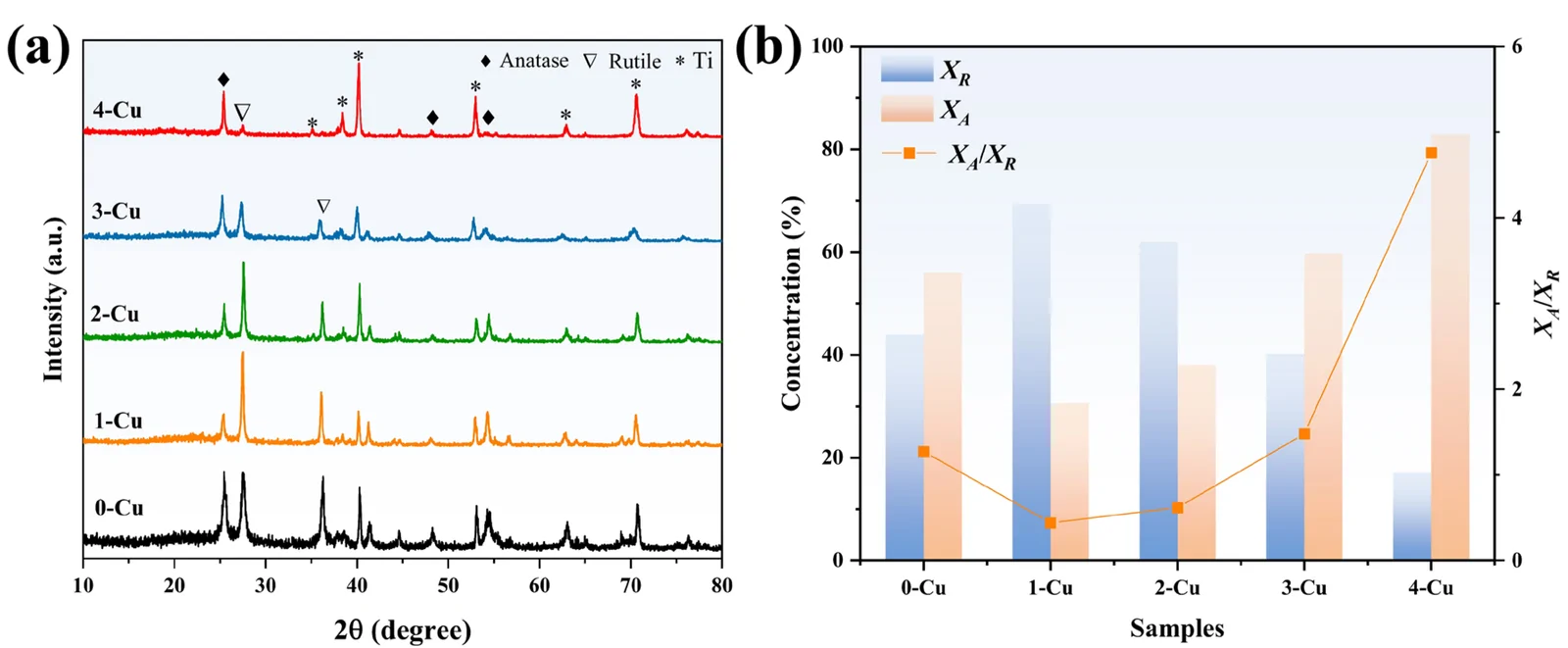
Phase analysis of MAO coatings containing different concentrations of Cu_2_O nanoparticles: (**a**) XRD patterns; (**b**) content of anatase and rutile phases.

The XRD analysis results of MAO coatings prepared with different concentrations of Cu_2_O nanoparticles are shown in Figure 7a. After the addition of Cu_2_O nanoparticles, the phase composition of the coating remained unchanged. This was because the concentration of Cu_2_O in the coating was below the detection limit of the XRD analysis [13]. The main phases of the coating were TiO_2_ and titanium. The TiO_2_ phase consisted primarily of anatase (A-TiO_2_) and rutile (R-TiO_2_). The mass fractions of A-TiO_2_ and R-TiO_2_ in each coating were calculated using the Spurr and Myers Equation (2) [29]:

(2) $$X_{R}=\frac{1}{1+1.26(\frac{I_{A}}{I_{R}})}$$

*X*_A_ = 1 − *X*_R_ (3)

where *X*_R_ and *X*_A_ represent the percentage contents of R-TiO_2_ and A-TiO_2_, respectively, *I*_R_ and *I*_A_ represent the integrated diffraction peak intensities of the R-TiO_2_ (110) crystal plane and the A-TiO_2_ (101) crystal plane, respectively. Figure 7b shows the content distribution of R-TiO_2_ and A-TiO_2_. As the particle concentration increased, the R-TiO_2_ content first increased and then decreased. The R-TiO_2_ to A-TiO_2_ content ratios were as follows: *X*_A_/*X*_R_ = 1.27 (0-Cu), *X*_A_/*X*_R_ = 0.44 (1-Cu), *X*_A_/*X*_R_ = 0.62 (2-Cu), *X*_A_/*X*_R_ = 1.48 (3-Cu), and *X*_A_/*X*_R_ = 4.76 (4-Cu). With the addition of Cu_2_O, the *X*_A_/*X*_R_ ratio first decreases and then increases. This indicated that adding an appropriate amount of nanoparticles (1–2 g/L) to the electrolyte induces a phase transition from A-TiO_2_ to R-TiO_2_ in the growing MAO coating. R-TiO_2_ is the high-temperature stable phase of TiO_2_ [8,30]. During the MAO process, the addition of nanoparticles at an appropriate concentration (1–2 g/L) increased the applied voltage and enhanced microdischarge intensity, thereby raising the local temperature and promoting the transformation of A-TiO_2_ to R-TiO_2_ in the coating.

**Figure 8:**
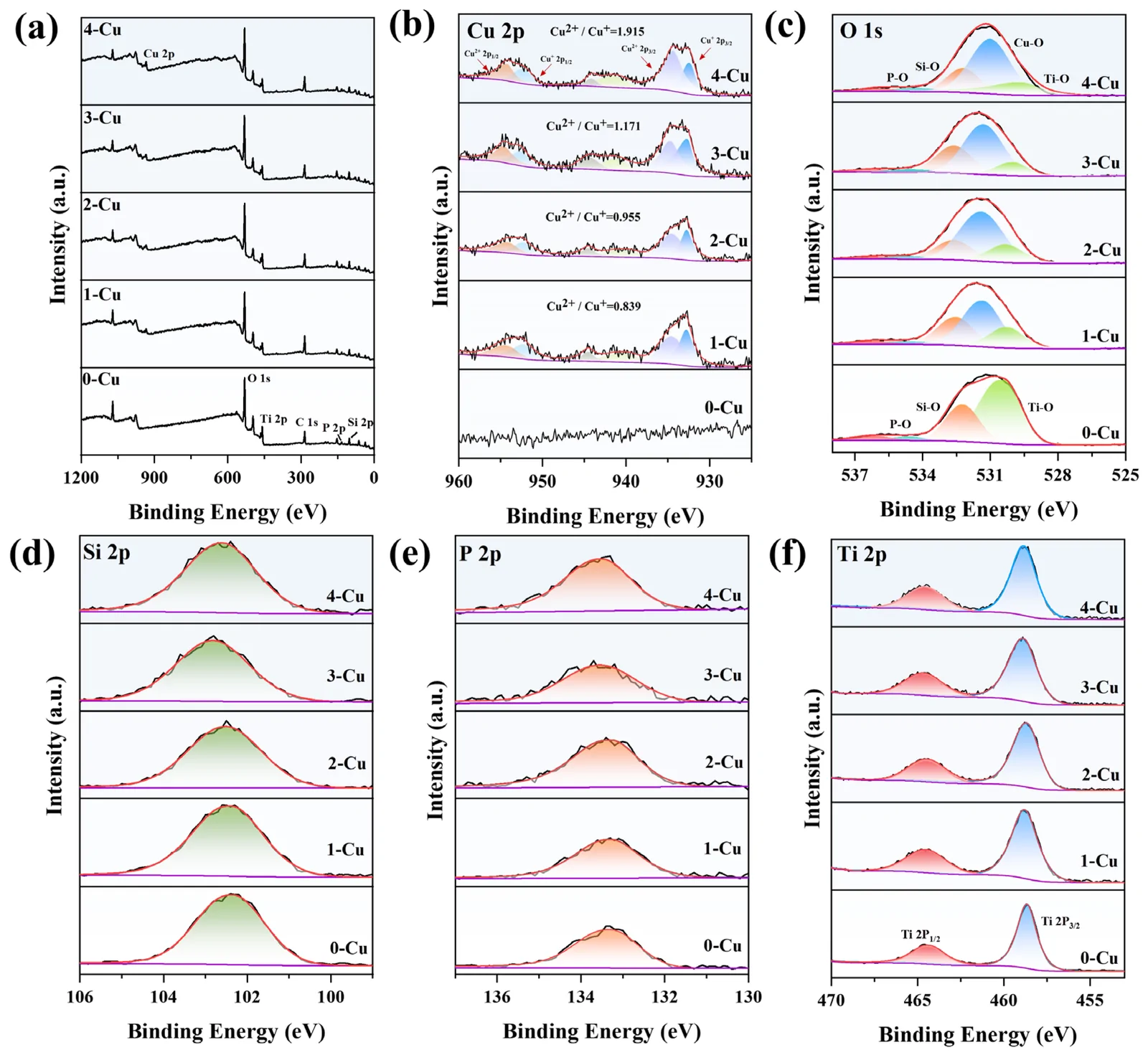
XPS spectra of MAO coatings with different concentrations of Cu_2_O nanoparticles: (**a**) full survey spectrum, (**b**) Cu 2p high-resolution spectra, (**c**) O 1s high-resolution spectra, (**d**) Si 2p high-resolution spectra, (**e**) P 2p high-resolution spectra, (**f**) Ti 2p high-resolution spectra.

Figure 8 shows the XPS spectra of MAO coatings. As shown in the full spectrum in Figure 8a, the surface of the 0-Cu coating primarily contained Ti, O, Si, and P. The full spectrum of the coating prepared with added Cu_2_O nanoparticles contained Cu, Ti, O, Si, and P. The chemical states on the coating surface were further examined using high-resolution spectroscopy. The high-resolution Cu 2p spectrum is shown in Figure 8b. For the coating prepared by adding Cu_2_O nanoparticles, the high-resolution Cu 2p XPS spectrum consisted of four characteristic peaks. The peaks at 952.4 ± 0.2 eV and 932.7 ± 0.1 eV corresponded to the Cu^+^ 2p_1/2_ and Cu^+^ 2p_3/2_ peaks, respectively, indicating the presence of Cu_2_O on the surface. The characteristic peaks at 954.3 ± 0.2 eV and 934.5 ± 0.2 eV corresponded to the Cu^2+^ 2p_1/2_ and Cu^2+^ 2p_3/2_ peaks, respectively, indicating the presence of CuO on the surface [31,32]. This indicated that Cu in the MAO coatings primarily existed in the forms of Cu_2_O and CuO. The forms of Cu in the coating were the same, but their relative contents differed. As the concentration of Cu_2_O in the electrolyte increased, the Cu^2+^/Cu^+^ ratio in the coating increased. The high-resolution O 1s spectrum is shown in Figure 8c, the Cu-containing coating could be deconvoluted into Ti–O (529.8 eV), Cu–O (531.2 eV), Si–O (532.4 eV), and P–O (534.5 eV) [33]. The binding energies of Si, P, and Ti in the 0-Cu coating and the coating prepared with added Cu_2_O particles remained similar. The high-resolution spectrum of Si (Figure 8d) showed a Si 2p binding energy of 102.3 ± 0.2 eV, corresponding to Si^4+^ [34,35]. In the P 2p spectrum (Figure 8e), the peak at 133.5 ± 0.1 eV could be attributed to phosphate compounds [36]. Figure 8f shows the high-resolution spectrum of Ti. The binding energies of the two Ti 2p peaks were 458.7 ± 0.1 eV and 464.5 ± 0.1 eV, corresponding to the Ti 2p_3/2_ and Ti 2p_1/2_ states of TiO_2_ [31].

**Figure 9:**
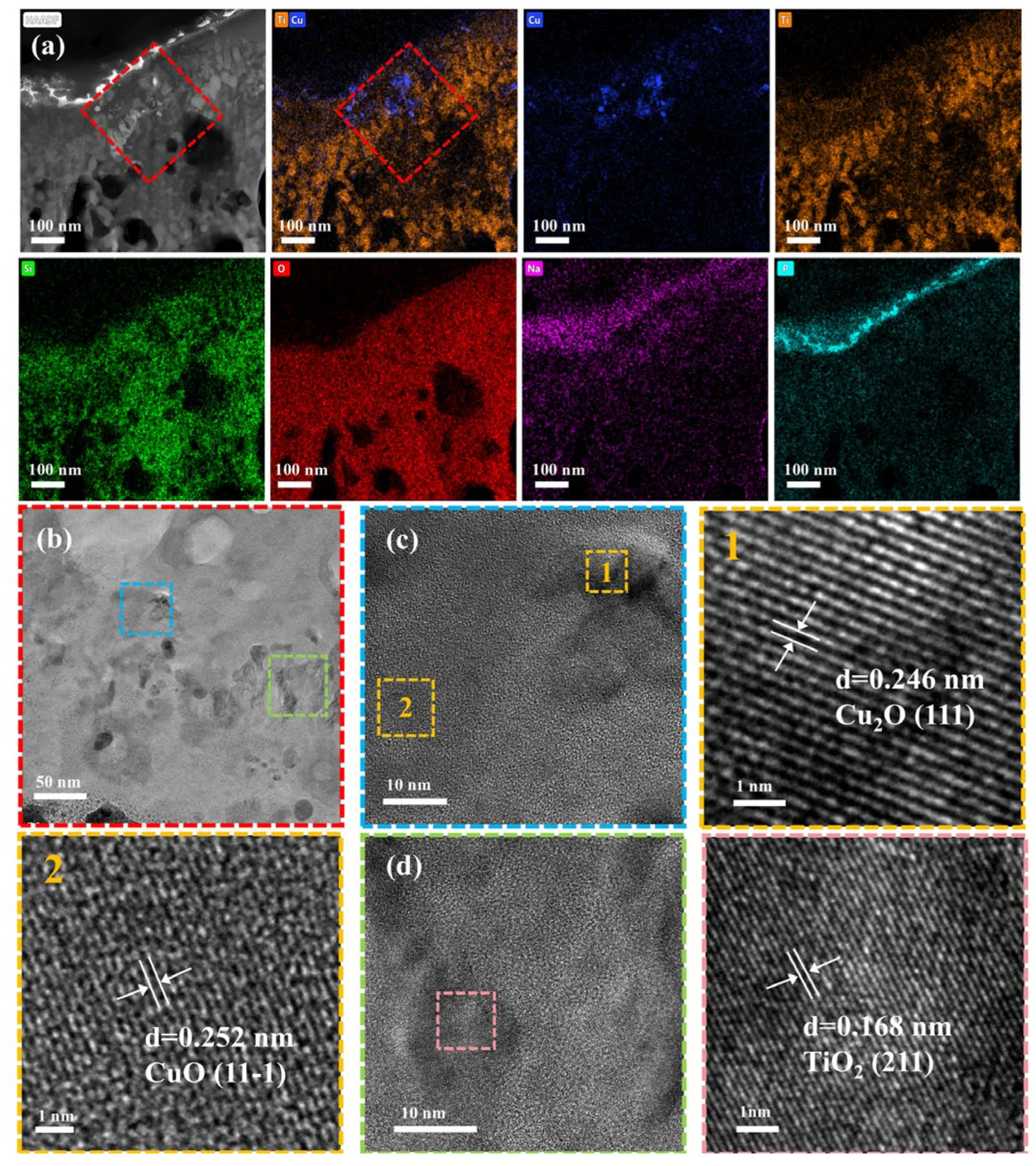
Cross-sectional TEM images of the 3-Cu coating: (**a**) EDS elemental maps; (**b**) low-magnification TEM images of two regions; (**c**) HR-TEM image of the Cu-enriched region; (**d**) HR-TEM image of the Ti-enriched region.

The surface of the 3-Cu coating did not exhibit the non-uniform morphology observed on the 4-Cu coating (Figure 2b), and it contained a higher Cu content (Table 1). To further investigate the morphology, crystal structure, and elemental distribution of the coating, a 3-Cu coating sample was selected for TEM analysis. Figure 9 shows a cross-sectional TEM image of the 3-Cu coating. EDS analysis revealed the presence of Cu, Ti, Si, O, Na, and P in the coating. The red box in Figure 9a is magnified in Figure 9b. Two regions with high concentrations of Cu and Ti, respectively, were selected for analysis. Figure 9b shows low-magnification TEM images of these two regions. Figure 9c presents an HR-TEM image of the region with high Cu concentration. HR-TEM observations of two randomly selected regions revealed distinct crystal structures. In Region 1, only Cu_2_O particles with (111) crystal planes were observed. No Cu_2_O particles with (110) crystal planes were found in this region (Figure 1). This observation suggested that the high-temperature and high-pressure environment inside the discharge channel may have influenced the particles. These extreme conditions transformed the surface of the Cu_2_O nanoparticles into the lower-energy (111) crystal plane. The TEM image obtained from region 2 showed a CuO phase with (11-1) crystal plane orientation. This finding indicated that during the MAO process, part of the Cu_2_O was oxidized to CuO. This result was consistent with the XPS results (Figure 8b). Figure 9d shows the HR-TEM image of the Ti-enriched region. This region was identified as the Ti_2_O phase based on the interplanar spacing.

### 3.4. Formation Mechanism of the Coatings

**Figure 10:**
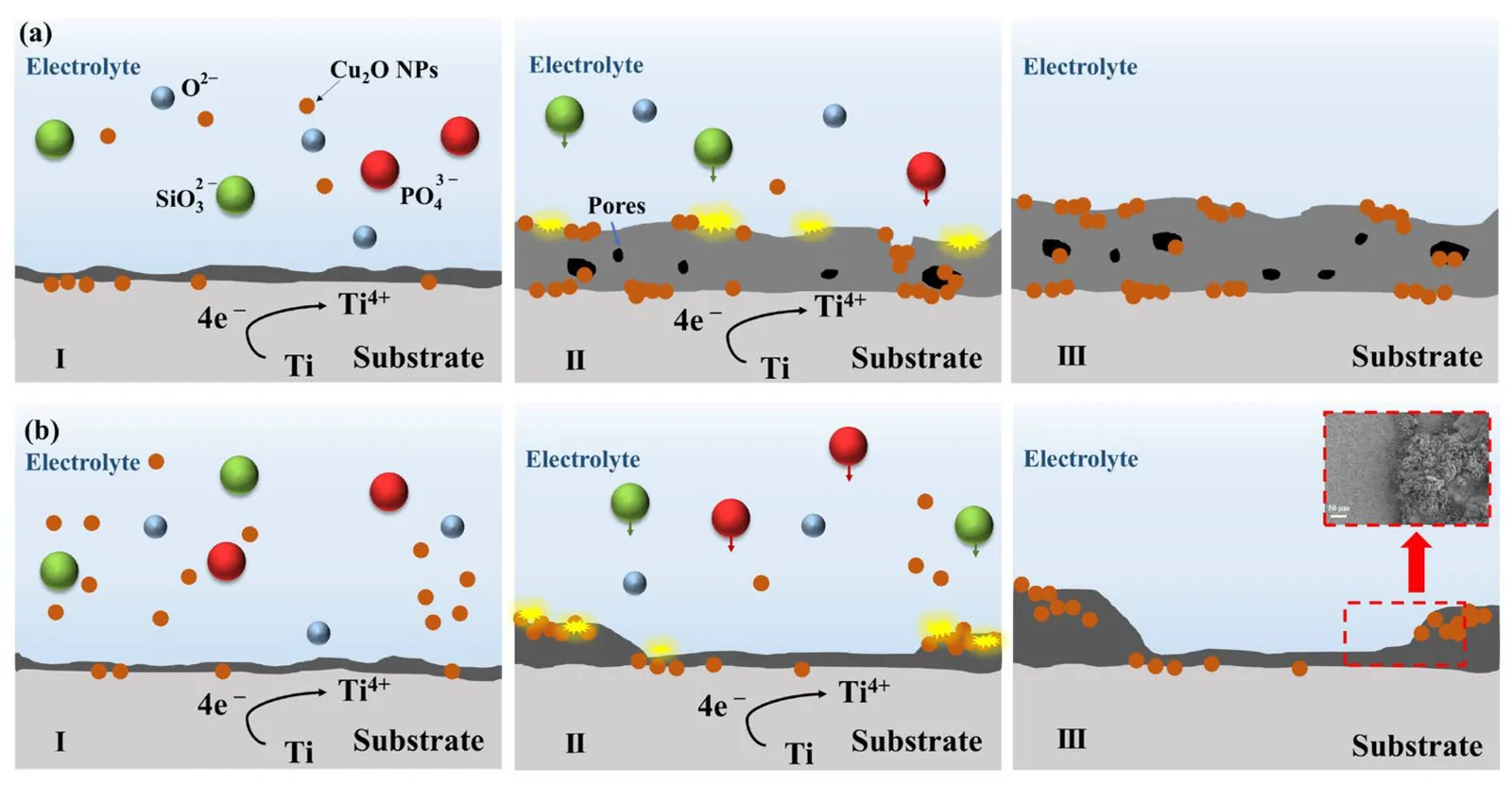
Schematic illustration of the incorporation mechanism of Cu_2_O nanoparticles into MAO coatings. (**a**) 3-Cu: (I) initial stage of MAO; (II) vigorous reaction during the MAO process; (III) uniform coating structure. (**b**) 4-Cu: (I) initial stage of MAO; (II) vigorous reaction during the MAO process; (III) non-uniform coating structure.

The coating prepared with 3 g/L of Cu_2_O nanoparticles exhibited a high Cu content and a relatively uniform surface. Therefore, this coating was selected for analysis of the particle incorporation mechanism. Figure 10a illustrates the growth process of the coating during the MAO process. It has been reported that in the early stage of MAO, anodic oxidation dominates, and Cu_2_O nanoparticles undergo directional migration toward the Ti substrate driven by the electric field force [37,38,39]. Meanwhile, during the growth of the oxide layer, nanoparticles may adhere to the surface through electrostatic attraction and van der Waals forces, thereby pre-forming a nanoparticle-enriched region at the metal/oxide layer interface. Figure 5 shows that a large number of enriched nanoparticles exist at the metal/oxide layer interface of the 3-Cu coating. When the voltage reaches the breakdown voltage, transient plasma discharge channels are excited by local dielectric failure, and spark discharge leads to local melting and sintering. The porosity of the 3-Cu coating is reduced by 8.36% compared with that of the 0-Cu coating (Figure 4). Some researchers have proposed that the high temperature of micro-discharges melts the nanoparticles, which then cover and plug the micro-channels [40]. It is speculated in this study that some nanoparticles may be drawn into the discharge channels and, during rapid solidification, melt together with the substrate and the oxide film to form the coating, thereby plugging the discharge channels and effectively reducing porosity. Meanwhile, the SEM and TEM cross-sectional morphologies of the 3-Cu coating in Figure 5 and Figure 9 reveal that there are numerous Cu-enriched bright regions on the surface layer, which are discrete nanoparticle distribution areas. The high temperature of micro-discharges causes local evaporation of the electrolyte, thereby causing the nanoparticles to adhere to the coating surface [41]. In summary, Cu_2_O nanoparticles are mainly incorporated into the coating under the combined action of electrophoretic force and micro-discharges.

Figure 10b shows the formation mechanism diagram of the 4-Cu coating. When nanoparticles are added in excess, the concentration of Cu_2_O nanoparticles in the electrolyte is relatively high. In the early stage of MAO, Cu_2_O nanoparticles still undergo directional migration toward the Ti substrate driven by the electric field force, and the voltage–time curve shows a linear increase in voltage with time (Figure 2a). At this stage, nanoparticles form a nanoparticle-enriched region at the metal/oxide layer interface (Figure 5e). However, as the voltage continues to increase, due to the edge effect, the charge density in the edge region of the Ti substrate is higher and non-uniform [42,43,44]. Most of the Cu_2_O nanoparticles preferentially attach to the edge of the Ti substrate, but this region cannot melt a large number of Cu_2_O nanoparticles in time, thus causing particle aggregation. The aggregated conductive particles in turn reduce the local resistance, resulting in a sudden drop in voltage (Figure 2a), which promotes dielectric breakdown of the oxide layer, leading to preferential discharge. The discharge becomes more concentrated, and a large number of Cu_2_O nanoparticles aggregate in the edge region (Figure 2b).

### 3.5. Antibacterial Performance

**Figure 11:**
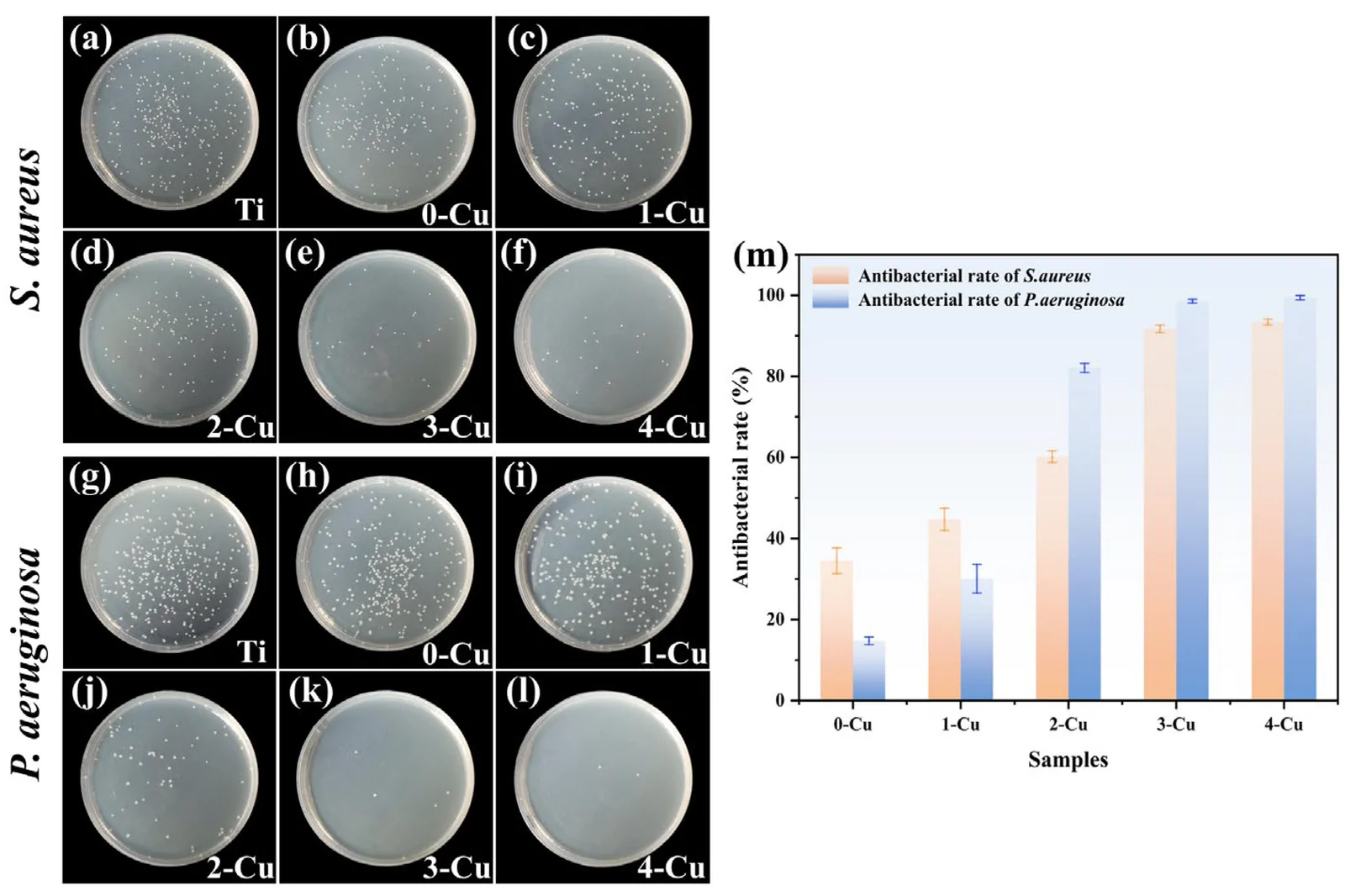
Antibacterial performance of coatings tested by the plate spreading method: (**a**–**f**) antibacterial performance against *S. aureus*; (**g**–**l**) antibacterial performance against *P. aeruginosa*; (**m**) antibacterial rate.

**Figure 12:**
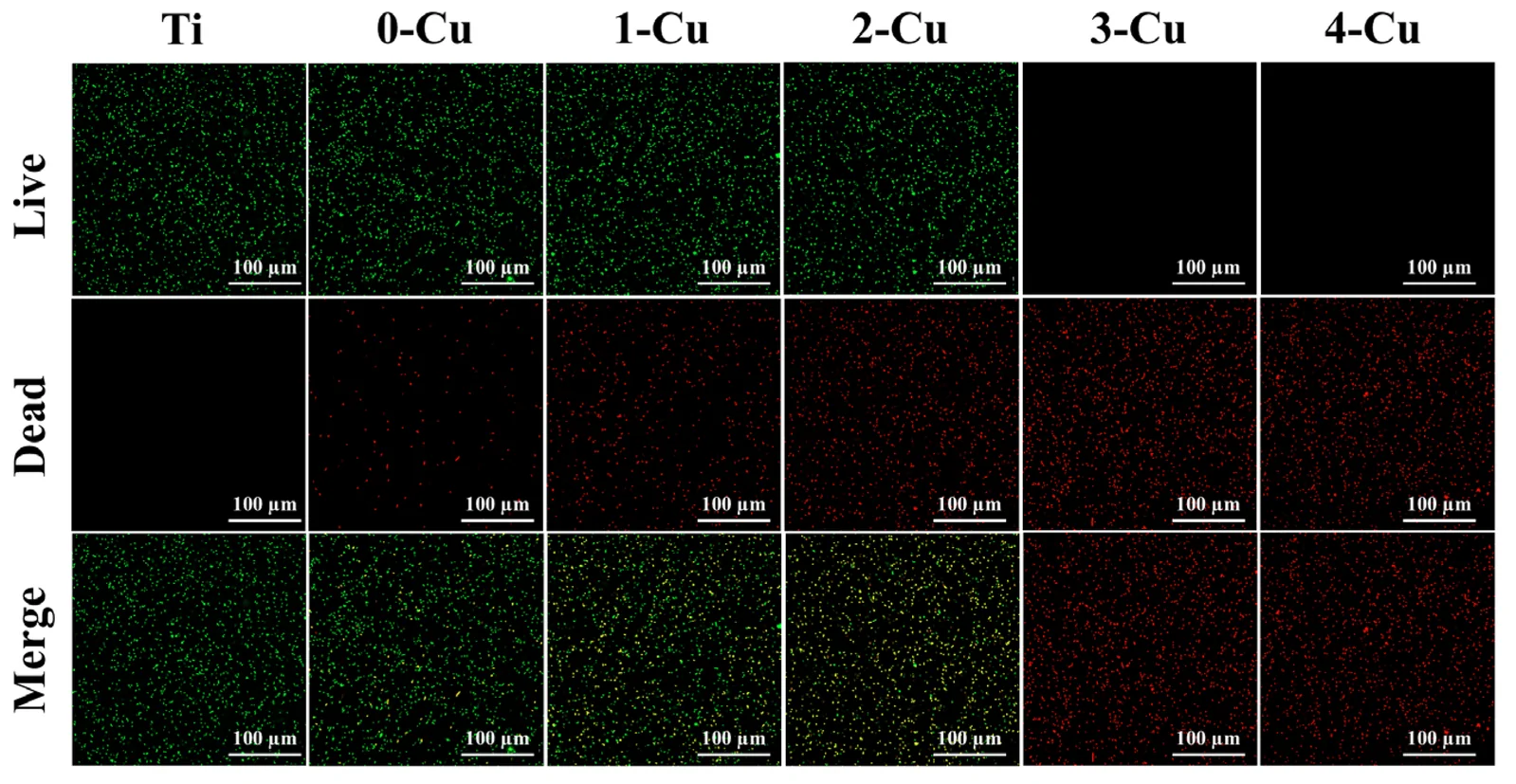
Viability staining analysis of *P. aeruginosa* after treatment with the coatings.

To investigate the antibacterial performance of MAO coatings with different concentrations of Cu_2_O nanoparticles, the coatings were co-cultured with *S. aureus* and *P. aeruginosa* for 24 h. The bacterial status in the cultures was observed. Figure 11 shows the antibacterial performance of the Ti blank control group and MAO coatings. The plate spreading method was used for this test. Numerous bacterial colonies of *S. aureus* and *P. aeruginosa* were observed on the Ti substrate. This observation confirmed that the Ti substrate lacked antibacterial performance. With increasing concentration of Cu_2_O nanoparticles, the inhibitory effect of the MAO coatings against *S. aureus* and *P. aeruginosa* was significantly enhanced. When 3 g/L and 4 g/L of Cu_2_O nanoparticles were added, only a few colonies of *S. aureus* and *P. aeruginosa* were observed on the 3-Cu and 4-Cu coatings. Figure 11m shows the antibacterial rates for different samples. For *S. aureus*, the antibacterial rates of the 3-Cu and 4-Cu coatings were 91.8% and 93.4%, respectively. For *P. aeruginosa*, the antibacterial rates of the 3-Cu and 4-Cu coatings were 98.57% and 99.43%, respectively. The antibacterial rates were significantly increased to over 90%. Therefore, these coatings qualified as effective antibacterial materials according to the national standard JC/T897-2014. The antibacterial effect of the coatings on adherent bacteria was further investigated using a live/dead bacterial staining method. Figure 12 shows the live/dead staining images of *P. aeruginosa* on the coatings. In these images, green fluorescence represented live bacterial colonies, and red fluorescence represented dead bacterial colonies [12]. The Ti substrate exhibited high-intensity green fluorescence. The 0-Cu, 1-Cu, and 2-Cu coatings began to show red fluorescence. The 3-Cu and 4-Cu coatings exhibited high-intensity red fluorescence. These results indicated that the 3-Cu and 4-Cu coatings prepared via the MAO process possessed stronger antibacterial capabilities.

**Figure 13:**
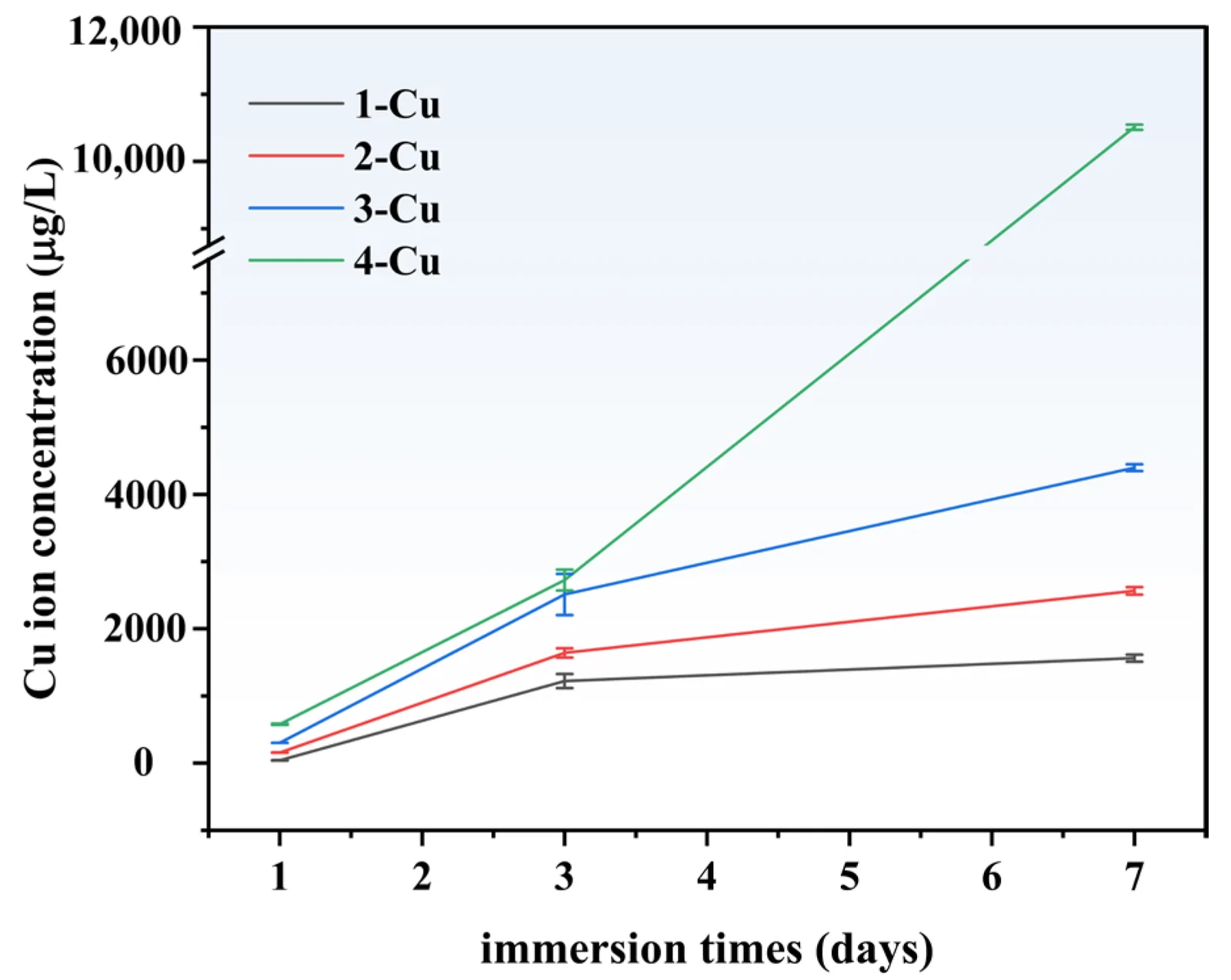
Cumulative concentration of released Cu ions.

The antibacterial performance of the coating was closely related to the release behavior of Cu ions [42]. Figure 13 shows the cumulative Cu ion release concentrations for MAO coatings containing different concentrations of Cu_2_O nanoparticles. The measurements were taken at 1, 3, and 7 days. From day 1 to day 3, the Cu ion release concentrations of all samples increased linearly. From day 3 to day 7, the Cu ion release rate for the 1-Cu, 2-Cu, and 3-Cu coatings was lower than the rate observed from day 1 to day 3. The 4-Cu coating continued to release a large amount of Cu ions. This occurred because a large number of Cu_2_O nanoparticles adhered extensively to its surface. The Cu ion release concentrations for the 3-Cu and 4-Cu coatings remained consistently higher than those for the 1-Cu and 2-Cu coatings.

### 3.6. Antibacterial Mechanism of the Coatings

During the MAO process, as the applied voltage increases, the initially formed oxide film undergoes dielectric breakdown, thereby generating micro-arc discharges. A single micro-arc discharge released a certain amount of energy. This energy is known as the average energy density per discharge (AEDPD). The AEDPD affected the local temperature during coating growth [45,46,47]. When a low concentration of Cu_2_O nanoparticles (1–2 g/L) was added, the nanoparticles blocked the micropores of the initial oxide film, increasing the local electrical resistance. The increase in local resistance made dielectric breakdown more difficult to occur. As a result, the operating voltage increased, and the AEDPD became higher. When the Cu_2_O nanoparticle concentration increased to 3 g/L and 4 g/L, a large amount of Cu_2_O nanoparticles was incorporated into the coating. The dielectric constant of Cu_2_O (approximately 12) is significantly lower than that of TiO_2_ (approximately 86) [48,49]. Materials with lower dielectric constants have a weaker ability to withstand the applied voltage before dielectric breakdown occurs. Therefore, the addition of a certain amount of Cu_2_O nanoparticles promoted micro-arc discharge. This promotion led to a decrease in AEDPD and, consequently, a decrease in local temperature. From a thermodynamic perspective, CuO tends to form at lower local temperatures [37]. Therefore, as the concentration of Cu_2_O nanoparticles increased, the Cu^2+^/Cu^+^ ratio gradually rose (Figure 8b).

Incorporating an appropriate concentration of Cu_2_O nanoparticles can achieve desirable antibacterial capability, and the antibacterial rates of the 3-Cu and 4-Cu coatings are higher than those of the 0-Cu, 1-Cu, and 2-Cu coatings (Figure 11). The live/dead staining images of *P. aeruginosa* show that the surfaces of the 3-Cu and 4-Cu coating samples exhibit a high level of red fluorescence intensity (Figure 12), indicating reduced bacterial viability on the 3-Cu and 4-Cu coatings. This is because Cu is considered an effective antibacterial agent [50]. Discrete copper nanoparticles are distributed on the coating surface (Figure 3). It has been reported that Cu_2_O and CuO can kill bacteria through contact killing, disrupting the bacterial cell wall and causing structural changes, thereby affecting bacterial morphology and function, and further altering the permeability of the bacterial cell membrane. Intracellular substances then begin to leak out, leading to bacterial death [51]. Therefore, contact killing may provide an auxiliary antibacterial effect for the coating. In addition, D. Yongabi et al. found that surface free energy is an important parameter controlling the short-term and long-term adhesion of eukaryotic and prokaryotic cells. High-polarity surface free energy surfaces such as SiO_2_ inhibit *E. coli* adhesion because *E. coli* itself has a high-polarity surface free energy, which produces strong electrostatic repulsion with high-polarity surfaces [52]. *S. aureus* and *P. aeruginosa* also possess relatively high surface free energy [53,54]. The coating may synergize with Cu ion killing by reducing the initial bacterial adhesion. It is worth noting that this study used ion release analysis as a preliminary indicator of antibacterial performance, and the determination of the contributions of contact killing and adhesion inhibition is mainly based on indirect results. In the future, direct verification should be carried out by observing bacterial morphology on the coating surface and investigating the adhesion behavior of bacteria on the coating surface.

Cu ions possess good antibacterial effects, and the antibacterial effects of copper species with different valence states have also been widely discussed. Cu^2+^ and Cu^+^ are two copper species with different valence states, and both have effective antibacterial effects [51,55]. The release of cu ions within 7 days was detected by ICP, and the results show that cu ions were continuously released over the 7 days, and the higher the concentration of ions released from the coating, the better the antibacterial effect of the coating. Therefore, the release of cu ions is the main antibacterial mechanism of the coating.

## 4. Conclusions

The incorporation of Cu_2_O nanoparticles into MAO coatings improved the surface microstructure and antibacterial performance of the coatings. The following conclusions were drawn:

(1) At an appropriate Cu_2_O loading, the nanoparticles were incorporated relatively uniformly into the coating. At excessive loading, the edge effect caused the incorporation to shift toward pronounced aggregation at the substrate edges.

(2) Cu_2_O nanoparticles were incorporated through combined electrophoretic and micro-sparks. Early fields drove Cu_2_O adsorption at the metal/oxide interface. At breakdown voltage, local heating fused some particles into the coating, leaving others discretely distributed on the surface.

(3) Coatings prepared with 3–4 g/L Cu_2_O nanoparticles demonstrated significant antibacterial activity. Copper ion release was the primary antibacterial mechanisms of the coating.

## Figures and Tables

**Table 1:** Elemental composition (wt.%) of the MAO coating surface.

Element (wt.%)	O	Na	Ti	P	Si	Cu
0-Cu	51.07 ± 0.12	0.86 ± 0.02	39.59 ± 0.10	0.97 ± 0.02	7.51 ± 0.03	0.00
1-Cu	53.26 ± 0.11	1.11 ± 0.03	28.49 ± 0.07	2.16 ± 0.02	13.54 ± 0.04	1.45 ± 0.03
2-Cu	52.79 ± 0.11	0.83 ± 0.03	29.61 ± 0.08	2.21 ± 0.02	12.89 ± 0.04	1.66 ± 0.04
3-Cu	54.10 ± 0.15	0.90 ± 0.04	28.79 ± 0.10	2.50 ± 0.03	11.44 ± 0.05	2.27 ± 0.05
4-Cu (center)	52.75 ± 0.15	0.67 ± 0.04	32.66 ± 0.11	3.24 ± 0.03	9.39 ± 0.04	1.30 ± 0.04
4-Cu (edge)	43.83 ± 0.13	1.62 ± 0.07	25.60 ± 0.02	1.46 ± 0.04	0.01 ± 0.08	27.48 ± 0.12

## Data Availability

The original contributions presented in this study are included in the article/Supplementary Material. Further inquiries can be directed to the corresponding author.

## Supplementary Materials

The following supporting information can be downloaded at: https://www.mdpi.com/article/10.3390/ma19204273/s1, Figure S1: Macroscopic photograph of the 4-Cu coating.
