# Supplementary material for: Formation and Antibacterial Mechanisms of Micro-Arc Oxidation Coatings on Ta2 Pure Titanium by Doping with Cu2O Nanoparticles
Source: Materials (Basel). 2026 Oct 9;19(20):4273. doi: 10.3390/ma19204273 (PMC13707075; doi:10.3390/ma19204273)
Supplement: Supplementary file 1 [file materials-19-04273-s001.zip › materials-4590343-supplementary.pdf]

Supporting Information

Formation and antibacterial mechanisms of micro-arc oxidation coatings on TA2 pure titanium by doping with Cu_2O nanoparticles

Xumin Zhang [1], Huie Hu [1], Ansheng Cheng [1] and Hao Wang [1,*]

1 Naval University of Engineering, Wuhan 430033, China
* Correspondence: 1920191001@nue.edu.cn

Supporting figures

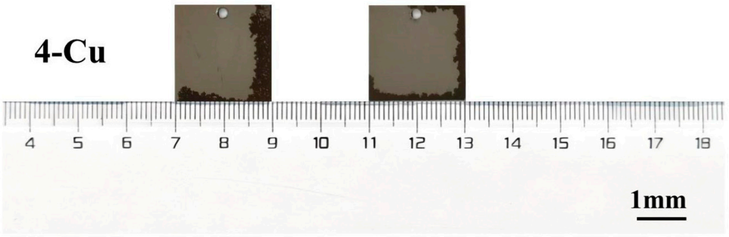

Figure S1. Macroscopic photograph of the 4-Cu coating.
